# BMX: Biological modelling and interface exchange

**DOI:** 10.1038/s41598-023-39150-1

**Published:** 2023-07-28

**Authors:** Bruce J. Palmer, Ann S. Almgren, Connah G. M. Johnson, Andrew T. Myers, William R. Cannon

**Affiliations:** 1 Physical and Computational Sciences Directorate, Pacific Northwest National Laboratory, Washington, USA; 2 Lawrence Berkeley National Laboratory, Berkeley, CA USA

**Keywords:** Software, Computer science, Computational models

## Abstract

High performance computing has a great potential to provide a range of significant benefits for investigating biological systems. These systems often present large modelling problems with many coupled subsystems, such as when studying colonies of bacteria cells. The aim to understand cell colonies has generated substantial interest as they can have strong economic and societal impacts through their roles in in industrial bioreactors and complex community structures, called biofilms, found in clinical settings. Investigating these communities through realistic models can rapidly exceed the capabilities of current serial software. Here, we introduce BMX, a software system developed for the high performance modelling of large cell communities by utilising GPU acceleration. BMX builds upon the AMRex adaptive mesh refinement package to efficiently model cell colony formation under realistic laboratory conditions. Using simple test scenarios with varying nutrient availability, we show that BMX is capable of correctly reproducing observed behavior of bacterial colonies on realistic time scales demonstrating a potential application of high performance computing to colony modelling. The open source software is available from the zenodo repository https://doi.org/10.5281/zenodo.8084270 under the BSD-2-Clause licence.

## Introduction

Biology provides a wealth of systems containing a very large number of individual agents performing a sequence of physical or chemical interactions. Collectively these groups of individuals build a complex system with the potential for emergent dynamics to form. The mechanism steps range from uncoupled or trivially coupled to highly complex with coupled feedback. The systems can span multiple length scales, from single molecule modelling to whole cell models. Simulations of large numbers of cells or detailed simulations of fewer cells can benefit from using high performance computing (HPC) resources to distribute the computation over multiple computing resources. Traditional HPC has focused on dividing calculations over CPUs, but the presence of relatively simple computational loops in many of these simulations suggests that they may benefit from distributing between GPU processors^1^. Stone et al.^2^ provided an overview of GPU use for molecular modelling and Zhou et al.^3^ utilised CUDA to simulate chemical reaction networks with their software *cuda-sim*. Higher scale problems were considered by Wilton and Szalay^4^ who increased whole-genome DNA sequence alignment speeds using GPUs while Thornburg et al.^5^ combined a range of different modelling modalities culminating in a GPU accelerated whole cell model. Here, we are interested in applying GPU acceleration to the simulation of a bacteria cell colony.

The cell colony is composed of the discrete cell agents and a shared continuous chemical environment. Each cell may by modelled as a subsystem with personalised growth dynamics, replication processes, and metabolic reactions. The cells interact with one another and their environment through mechanical force interactions (cell-cell and cell-environment) and nutrient transport exchange between cell and environment. Nutrients may also diffuse passively in the environment or be transported via convection. These interactions are important for cell community growth^6,7^ where the supply of vital nutrients to the cells can be affected by cell resource competition or varying nutrient diffusion rates^8^. Additionally, the cells in a colony are not static and each cell may move through the environment via active migration or passive cell-cell shunting.

A range of software has been developed to simulate cell colonies by viewing each individual cell as a separate agent and using agent based models (ABMs)^9^. Exemplar packages, including CompuCell3D^10^, PhysiCell^11^, and ChemChaste^12^, each emphasize and capture different aspects of a cell community model. These packages vary in their cell descriptions (how they model each individual cell agent; such as using the cellular automata (CA), or centre-based models^13^), mechanical descriptions (how the cells interact and move; such as Hookean spring forces or chemotactic motion), and environment descriptions (whether environment reactions and varying diffusion rates are considered). These descriptions become complex when simulating realistic colonies where simulations become computationally expensive in order to capture appropriate levels of detail. Therefore high performance computing methods become necessary for realistic colony modelling and GPU acceleration can provide even further performance gains^14^. However, these example packages are not configured to be readily accelerated to use GPUs and new packages which utilise high performance computing are under development.

Currently, progress has been made towards developing an understanding of cell and colony shape and cell growth biochemistry using high performance methods^15^. Sussman^16^ considered a vertex model approach through the *cellGPU* package aiming to efficiently compute the evolution of cell shape in connected tissues. Meanwhile, Cagigas-Muniz et al.^17^ used a CA model to demonstrate GPU acceleration techniques with Jelinek et al.^18^ applying a different CA model to simulate dendrite growth. Centre-based modelling approaches have been used to produce a high performance model specific to epithelial tissue morphogenesis^19^. However, these models have only considered simple environment conditions, if considering an environment at all.

Considering the cell models without an environment simplifies the problem description and implementation at the cost of a reduction in simulation capability, such as considering domain decomposition methods to partition the cell populations into sets for each CPU or GPU process^20^. Song and Lei^21^ provided an object-orientated implementation, *ParaCells*, that permits a range of cell descriptions, such as CA or centre-based models, and whose behaviors may be readily customised by a user. However the implementation is limited to the CUDA protocol, with the associated portability issues of architecture restriction, and only supports simple environment descriptions without chemical reactions or variable diffusion rates. *BioDynaMo*, introduced by Breitwieser et al.^22^, is a similarly modular software that considers cells in an environment, with simple chemical transport features describing constant diffusion rates and chemical decay, with equations solved using the central difference scheme. While these packages advance the progress of high performance ABMs, they do not capture the physics and chemistry of a growing cell colony in a high performance manner.

Here, we introduce BMX; a high performance, GPU accelerated, hybrid continuum-discrete approach to modeling the nutrient transport in growing 3D cell colonies^23^. In BMX, we are primarily concerned with building a high performance simulation capable of modelling the physics derived interactions of a large number of cells with a general environment model with variable nutrient diffusion rates. In our approach, each cell in the colony is allowed to maintain its own unique state as determined by its interactions with other cells and the environment. Cell growth is driven by the consumption of a nutrient species, taken in from the environment, and cell division is based on cells reaching a critical size. Cells interact mechanically with other cells through a set of soft forces that are repulsive at short distances and weakly attractive at longer distances. BMX was developed with a set of key capabilities in mind:

- Efficiently simulate a realistic (large) number of cells in a 3D cell colony
- Model nutrient transport through a cell colony with adaptable nutrient diffusion rates
- Simulate on real time scales with timescale partitioning
- Provide portable code not limited to a specific GPU architecture

BMX is written in C++ and supports exascale simulations of partial differential equations (PDEs) through building upon the AMReX adaptive mesh refinement software framework to implement a finite volume solver method^24^. The BMX code is derived from a previous application, MFIX, that was created on top of AMReX to simulate particle laden flows. AMReX supports massive parallelism and provides methods for performing domain decomposition, load balancing, sorting and binning of particles, particle-mesh interpolation, neighbor finding, reduction operations, and the parallel communication of particle data. Using the performance portability abstractions provided by AMReX, the code can run on NVIDIA, AMD, and Intel GPUs, and can take advantage of OpenMP acceleration for CPU-only execution, all in a single code base. Through the introduced capabilities, BMX supports the *in silico* modelling of realistic cell colonies. To demonstrate the simulation of a large number of interacting cells under realistic laboratory conditions we consider a series of small exemplar test cases based on the growth of a bacterial colony growing on an agar medium^25^. A number of simulations starting from a single cell were then run for an extended period of time. We show that, with the assistance of both CPU and GPU parallelization, BMX may simulate large cell systems vital for realistic modelling of colony dynamics within real time scales.

## Results

A colony of bacteria cells were simulated with a simple “ABC” metabolism (see Section “Methods” for details). The simulation model was composed of an initial progenitor cell placed on an agar support medium with the surface and cells exposed to the surrounding air. The same simulation setup was used to demonstrate the formation of a vertical column and horizontal spreading in bacteria cell colony growth and the phenomena of colony branching in diffusion limited nutrient environments where the results were visualised using ParaView (v5.10)^26^. We also considered the simulation scaling with respect to the time per simulation element and with the number of processing units, see Supplementary Information SI.1.

### Vertical column growth

**Figure 1:**
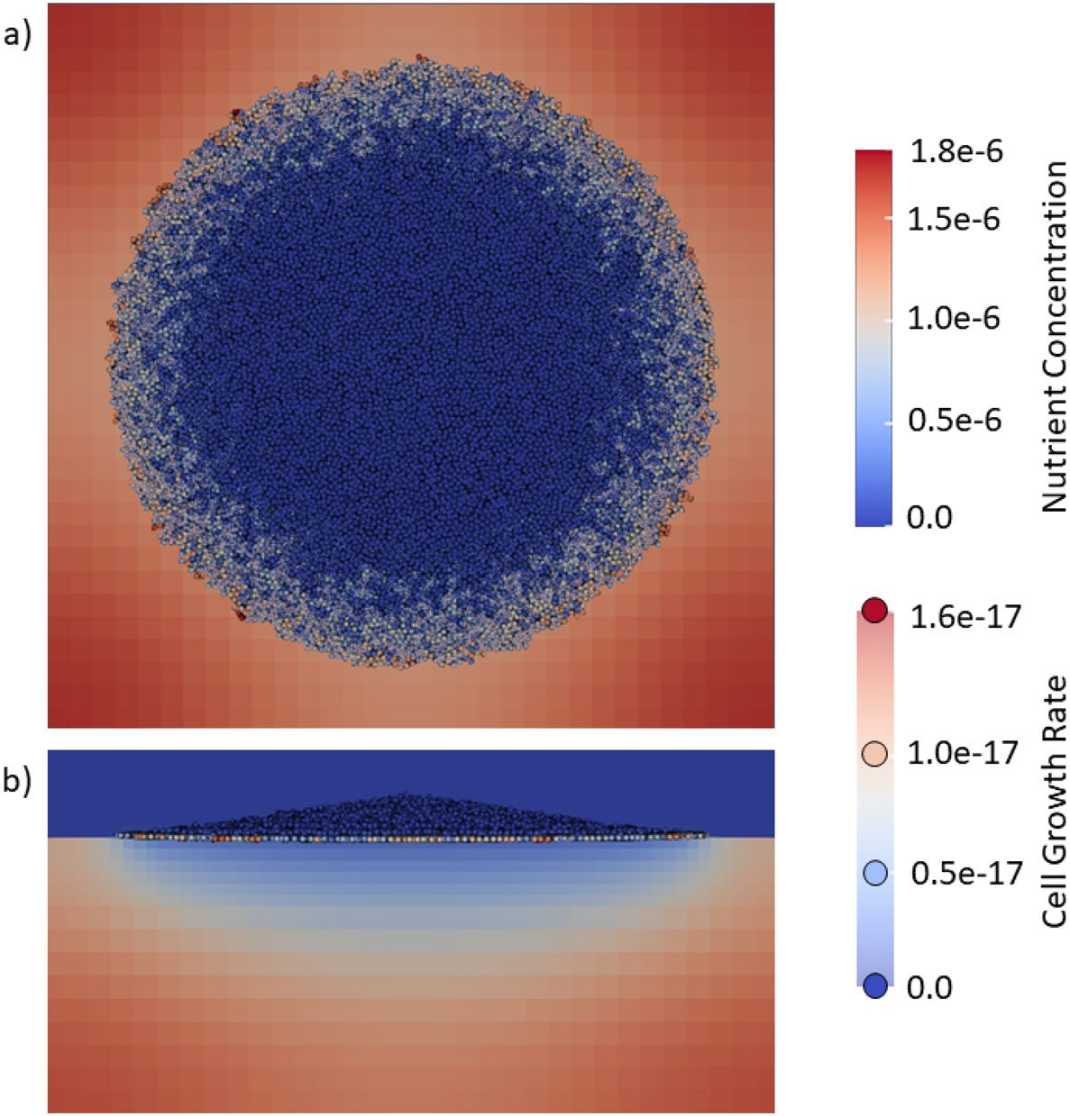
Graphic showing the particle configuration and the concentration of the environment nutrient species *A* at the surface of the growth medium after 200000 seconds. The particles are color coded by the volume growth rate ranging from blue (low) to red (high). The concentration of species *A* also uses the same color coding. (**a**) The simulation projection on the x-y plane showing the spreading of the colony over the agar. (**b**) projection in a plane passing vertically. This side-view shows the vertical growth of the colony and depletion of nutrients within the agar due to diffusion of nutrients to, and consumption by, the bacteria cells. The color coding is the same as in (**a**).

To simulate the formation of a bacteria colony we considered a model consisting of a $128\times 128\times 64$ $\mu$m system filled with agar growth medium up to the 48 $\mu$ level in the *z* direction. A single seed particle located on the surface of the growth medium at the center was used as an initial condition and the system was allowed to develop for 300000 steps, with each step representing 1 second of simulated time (the total simulation is about 3.5 days). The simulation grid consists of three levels; a fine level near the surface of the growth medium with 1 $\mu$m cubic grid cells and progressively coarser grid cells moving away from the growth surface (see "Methods" section for details). At the end of the simulation, the system contained 23567 particles, as may be seen in Fig. 1.

A visualization of the system viewed from above the growth medium surface is shown in Fig. 1a. The figure shows both the particles forming the colony cluster and the concentration of species *A* at the agar surface. The particles are color-coded by the volume growth rate, with the lighter colored particles near the edge showing higher grow rates than the darker particles at the center of the cluster. Figure 1b shows the same system from the side and one can clearly see a significant depletion regime below the growing cluster in which nutrient has disappeared. At the end of the 300000 seconds, the nutrient is depleted and the entire system is blue suggesting the system is not large enough to support continued growth. The role of nutrient depletion on colony growth is well known with nutrients limiting the spatial extent of the colony^27,28^. The side view of the particle cluster shows a slight peaking towards the center, and this vertical column growth is indicative of the morphology seen in many real-life biological systems^29^.

**Figure 2:**
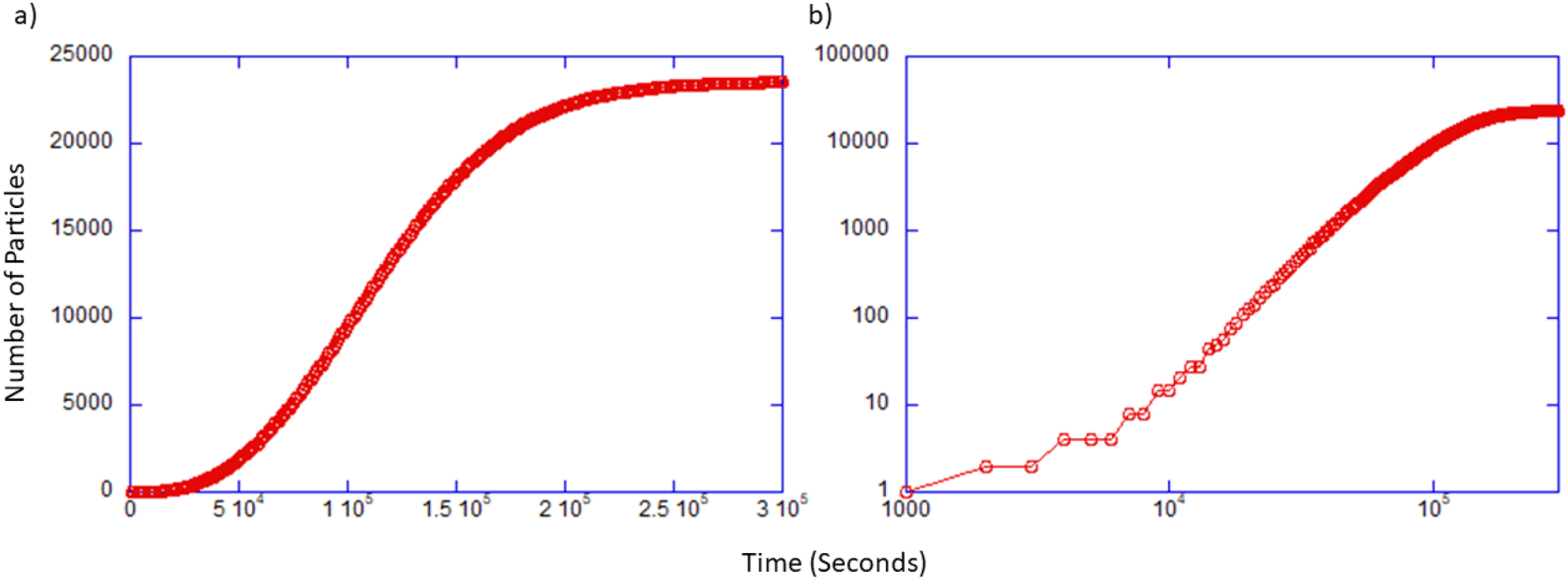
Plot of number of particles in large simulation as a function of time for the cell colony simulation shown in Fig. 1. (**a**) The particle (bacteria cells) count plotted on linear axis. (**b**) Plot of the number of particles plotted on log-log axis.

Plots for the number of particles as a function of time are shown in Fig. 2. When considering a linear plot, Fig. 2a, the particle count over time displays distinctly non-exponential behavior. The sigmoidal growth curve shows a rapid increase at short times but a gradual slowing down as the supply of nutrients is depleted. At intermediate times, there is an approximately linear regime, indicative of a diffusion limited growth constrained by the time it takes for nutrients to diffuse to the growing colony. A log-log plot of the same data, shown in Fig. 2b, also indicates a linear regime at intermediate times. Furthermore, as nutrients are depleted locally, the supply of nutrients to the growing cluster is limited by the diffusive flux of nutrient from exterior regions. In analogy with an absorbing sphere in a three-dimensional infinite medium^30^, the flux should be asymptotically constant and this would lead to a constant growth rate. At later times the entire simulation volume is depleted in nutrients and growth is restricted not just by diffusion but also by the exhaustion of the nutrient source.

### Colony branching

**Figure 3:**
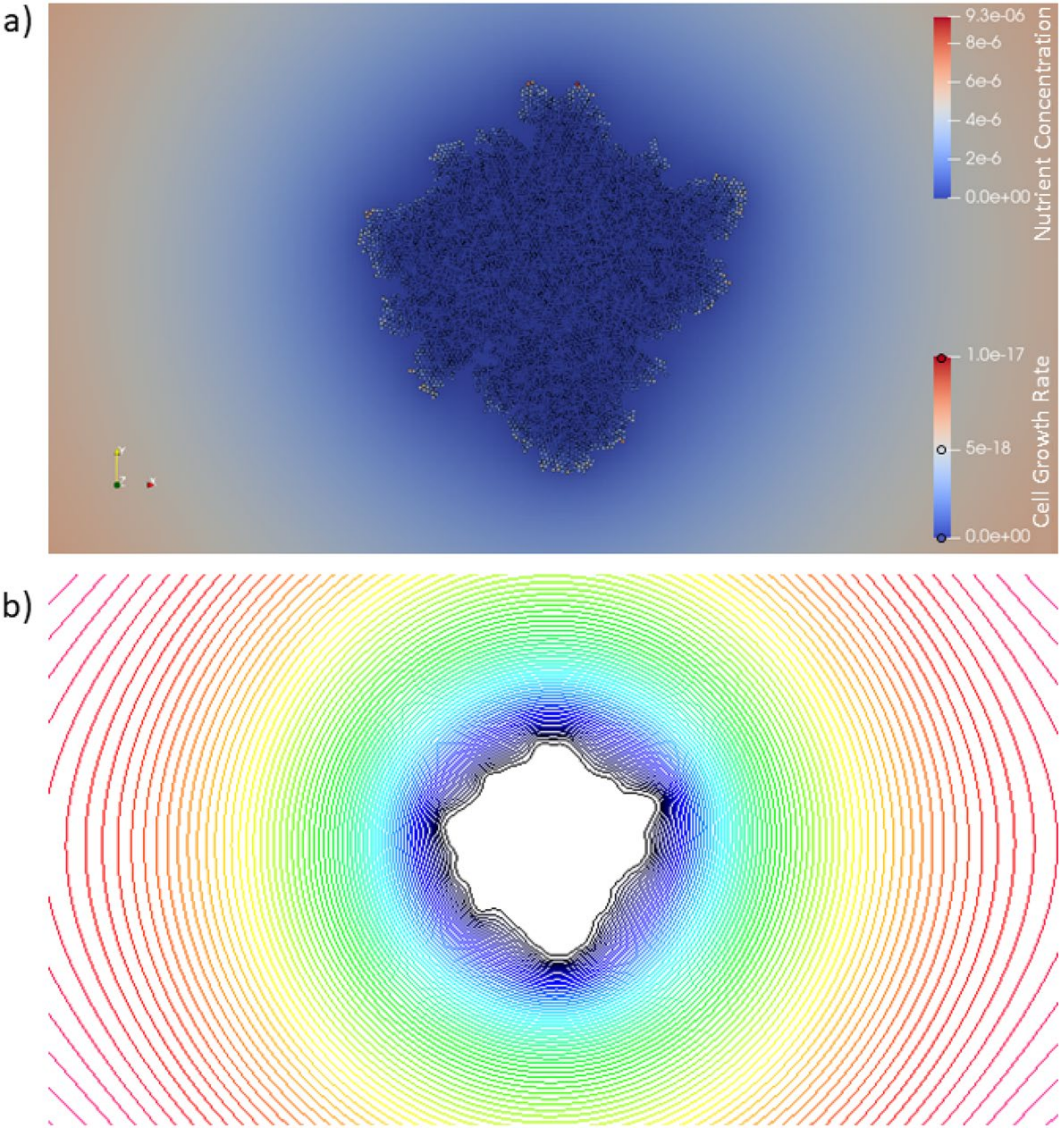
Limiting the nutrient diffusion rate by the number of bacterial particles can affect the colony configuration. (**a**) Graphic showing the final particle configuration and the concentration of the nutrient species in a plane at the surface of the agar growth medium for growth on a very thin medium. (**b**) Contour plot of the nutrient concentration just below the growth surface for the cluster shown in (**a**). Black corresponds to low concentration and red corresponds to higher concentrations.

It has been well known for some time that diffusion-limited growth in two dimensions is morphologically unstable and, in the absence of stabilizing features such as surface tension or surface diffusion, will result in branched, fractal-dimensioned structures^31,32^. The branching contributes to a rough external boundary to the bacteria colony where variations in access to environment nutrients between peaks and troughs affect the growth rate of the different morphological structures. This instability was originally observed in a simple lattice model developed by Witten and Sander^33^ and subsequently investigated in depth by Meakin^34^. Inspired by this work, a model was generated that would produce the branch structures seen in simulations of diffusion-limited aggregation (DLA). A system was prepared of dimensions $512\times 512\times 6$ $\mu$m. The main difference between the branching simulation and the vertical growth simulation is that the growth medium was reduced to 1 $\mu$m thick and is represented by one grid cell in the vertical direction. For this simulation, only one level of grid cell sizes were used and the nutrients are constrained to reach the growing cluster only by diffusion from the edges. This resulted in slower growth than for the thicker agar growth medium shown in Fig. 1, where nutrients can also reach the cluster by diffusing up from below. The final cluster is show in Fig. 3.

As shown in Fig. 3, the distinctive heterogeneous growth is more active at the tips of branches (seen as lighter color particles in the active growth regions) and less active in more shielded regions of the interface that are located in interior parts of the cluster. This occurs because these interior regions are screened by the outer branches and nutrients do not have a chance to penetrate far into the cluster^35^. Although the cluster shown in Fig. 3a is at best irregular, it is reasonable to suppose that if the simulation were allowed to proceed for a much longer time that it would become visibly branched as well. Other recent modeling efforts based on continuum equations and assuming a 2-dimensional geometry have shown similar results^36^ and are in accord with experimental observations. A contour plot for the nutrient concentration field of component *A* just below the surface of the growth medium, shown in Fig. 3b, shows fluctuations that mimic the outline of the growing cluster. The contours show that the density of contour lines is slightly higher around outward-facing projections and slightly lower around inward-facing intrusions. These variations in the density of contour lines correspond to variations in the concentration gradients and hence, in the flux of nutrient to the colony surface.

## Discussion

BMX was developed to produce realistic, large scale, simulations of 3D cell colony growth within real time frames. To this aim we utilised CPUs coupled with GPU acceleration using the AMRex package to provide a portable high performance simulation suite. Additional capabilities were added to the AMRex base to handle the dynamics of cell “particles” with the inclusion of associated interaction force laws and cellular metabolic models.

To test the software we considered two cases of bacteria colony growth with known dynamics; vertical colony formation in nutrient rich conditions, and branching and colony roughening in diffusion limited nutrient conditions. By changing the simulation domain to use a thicker and thinner agar layer both of the phenomena could be qualitatively reproduced without the need to change the metabolic models for the bacteria particles. The expected results were produced, both in colony shape and particle count for a system of bacterial cells utilizing a simple “ABC” metabolic model. The simulations showed the expected vertical column formation and horizontal spreading that is expected from simple cell interactions and nutrient diffusion in colonies as shown by^25^. The same simulation model was also able to reproduce branching and roughening of the bacteria colony boundary when the amount of agar support medium was reduced as expected.

The simulations presented in this article considered a simple “ABC” model for the bacteria cell metabolism. This model may be readily changed by a user to incorporate a more complex, and realistic, cell model to determine the behavior of the simulation particles. BMX was designed to be modular and readily adaptable to consider different biological models. Future work on the underlying software would look to expanding the capabilities and usability for user with more experimental, rather than programming, interest. The size and shape of a particle can affect the growth of a cell colony and adding new cell shapes and sizes can expand the space of possible models. For example, fungal and filamentous bacterial models may be implemented by considering elongated or cylindrical particles with the possibility of modelling budding yeast by providing asymmetric particle capabilities. Complex particle shapes necessitate the addition of further force laws to capture the effects of the varying interaction geometries. New force laws may also be provided for further particle-particle interactions and cell motilities. The expansion of the set of interactions is facilitated through the additive Hamiltonian formulation used in BMX, see section “Particle Model”.

More complex biological systems, such as biofilms and bioreactor systems, can include multiple cell species and chemical reactions and cell-cell chemical signalling in the environment. These systems may be considered by expanding the transport and chemistry models provided within the BMX examples. The diffusion aspects and the inclusion of advection processes, such as providing a directed flow through the system, may be implemented by following the BMX approach for the effective diffusion rates. Rates may be made to be dependent on the state of the environment, such as chemical concentrations in the grid cell, as a model for cell extracellular matrix excretion or advective flow for microfluidic modelling. The implementation of chemical reactions in the environment can support modelling of particle-particle signalling and environment degradation studies. This expansion would require the implementation of reaction-diffusion PDEs that would provide an additional source/sink term to the BMX diffusion equation (see Eq. 1). The inclusion of more complex environment PDEs is facilitated by the coupling to the AMRex suite as the underlying GPU acceleration is already provided.

In this paper we have presented BMX, a high performance agent based modelling software for real time modelling of realistic cell colonies. The introduction of our software fulfills a gap in the current capabilities and leverages high performance computing facilities towards large scale modelling of biological systems. The code may be readily adapted by a user to consider a range of cellular systems and we envisage the adoption of BMX for modelling both bacterial systems. Overall, BMX presents a great step towards the efficient high performance modelling of realistic biological systems.

## Methods

A BMX simulation consists of the domain volume that contains a transport model on a meshed grid and a collection of particles that represent the bacteria cell colony. The user can change how the particles interact with themselves and the environment through both a mechanical force model and a metabolic model controlling the growth and division of bacteria particles. Coupling between the fluid domain and the particles is performed through the exchange of nutrients between the two systems, requiring the nutrients to be included in the both the particle and environment subsystem descriptions. BMX has been developed with a modular design in mind so that these different aspects of a BMX simulation may be modified separately to assist model development. Currently, reactions only occur within biological cells, but the models could be extended to include reactions in the fluid environment.

The following discussion will make reference to both grid cells on the computational ‘cell’ will refer to cells on the computational grid and ‘particles’ will refer to biological cells.

### Transport model

**Figure 4:**
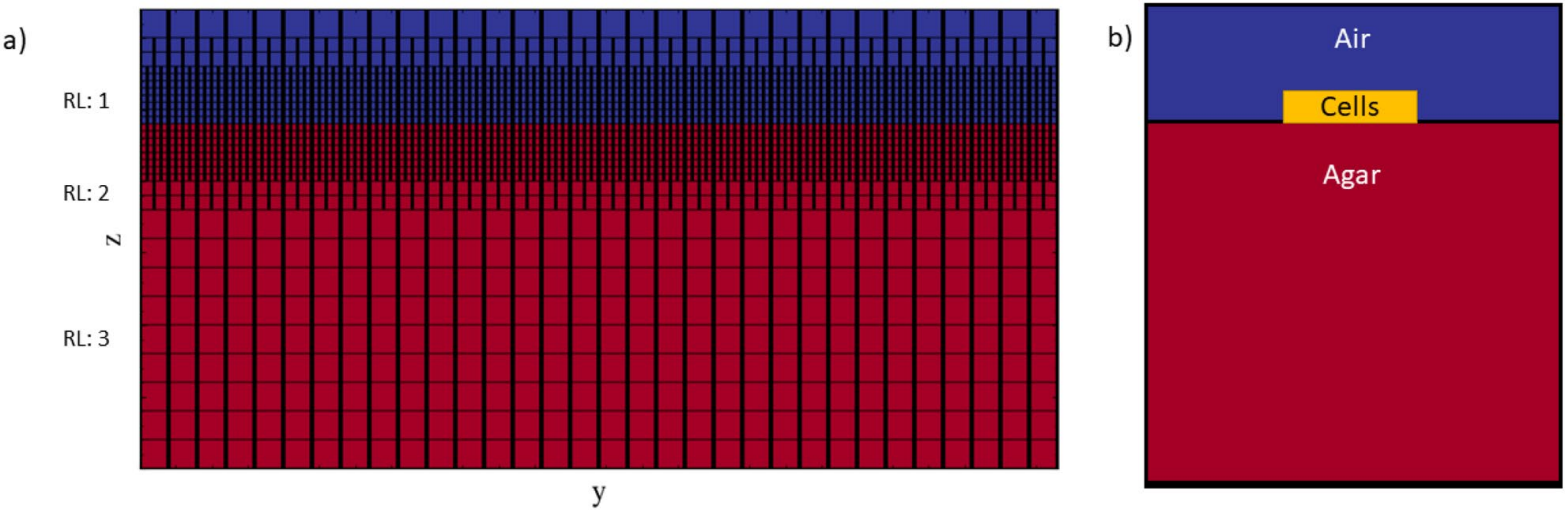
A mesh is applied over the model domain with a range of refinement levels for computational efficiency. (**a**) AMReX grid showing three levels of refinement above base level. The agar support medium is shown in red and the air region is shown in blue. The refinement levels were selected to produce a finer mesh towards the air-agar interface where strong variations may be expected. (**b**) Cartoon showing the simulation set up where a single bacteria cell particle is initialised in the center of the interface between the air region and the agar support.

In this article we apply BMX to simulate the growth of a multicellular aggregate on a layer of agar support medium that is saturated with a nutrient solution. The simulation volume is a 3D rectangular domain where the agar surface is parallel to the *xy* plane and perpendicular to the *z* axis with the remaining volume composed of air. The system is periodic in the *x* and *y* directions and has Neumann (zero flux) boundary conditions on the upper and lower surfaces. The air-agar interface is located somewhere between the upper and lower surfaces. The depth of agar provides a finite reservoir of the nutrients. We discretize this physical system using a block-structured hierarchical mesh, where at each level of refinement we have a union of rectangular boxes with a refinement ratio of 2, as shown in Fig. 4. The highest level of refinement is chosen to correspond to grid cells that are approximately the same maximal volume as a single particle on the threshold of division. The refinement is chosen to be highest near the agar-air interface since that is where the biological cells live and grow, as shown in Fig. 4b. The coarsest mesh is limited by the size of the system and the minimum size of the gradients of the field variables. At present, these refinement levels are fixed at the start of the calculation.

The transport of nutrients is governed by the set of diffusion equations

1 $$\begin{aligned} \frac{dc_i}{dt} = -D\nabla c_i +S_i({{\textbf{C}}}) \end{aligned}$$

where $c_i$ is the concentration of the *i*th dissolved species in the vector ${\textbf{C}}$, $D_i$ is the corresponding diffusion coefficient and $S_i(\textbf{C})$ is a spatially varying source or sink term for $c_i$. $S({{\textbf{C}}})$ is governed by the presence of particles and the reactions inside the particles. If there are no particles in a grid cell, then $S({{\textbf{C}}})$ is zero for that grid cell. BMX utilises the AMRex package to solve Eq. 1 and can support more complex transport models that may be written by a user by editing the underlying BMX C++ code, including the implementation of reactions occurring in the environment.

In order for nutrients to diffuse into and out of a grid cell, the cell must contain water. All grid cells below the agar-air interface are assumed to contain water, and diffusion coefficients in these cells are set to $D_{0_i}$, the diffusion coefficient for the *i*th species in water. Grid cells above the agar-air interface that do not contain any particles are assumed to contain only air, and all diffusion coefficients in those cell are set to zero. We describe a grid cell as containing a particle if the (physical) center of the particle is located within the cell. The diffusion coefficient is influenced by the presence of the particle(s).

In BMX, particles are treated as occupying a finite volume of space that nutrient must diffuse around, thus impeding the free-diffusion of nutrients. To capture the effect that cells have on local diffusion rates we define effective diffusion coefficients, $D_{eff_i}$. We will use the results of Khirevich *et al.*^37^ on diffusion in beds of random packed spheres to develop a model for the extracellular diffusion coefficient. The effective diffusion coefficient in a bed of random packed spheres can be modeled by an equation of the form

$$\begin{aligned} D_{eff} = \frac{\phi }{\tau }D_0 \end{aligned}$$

where $\phi$ is the volume fraction of fluid (the volume fraction of spheres is $1-\phi$), $\tau$ is the tortuosity and $D_0$ is the diffusion coefficient in the unoccupied fluid. The tortuosity accounts for the fact that diffusing molecules may need to take a more winding path through a porous medium in order to spread, see Supplementary Information S.2 for details. We solve Eq. (1) on the multilevel mesh hierarchy using the linear solvers in the AMReX framework. Here, diffusion coefficients are associated with the center of the grid cell and averaged to cell faces for use in the linear system. If a grid cell on one side of a face has zero diffusion coefficient, the value of the coefficient on that face is set to zero to enforce no diffusion into the cell.

### Particle model

**Figure 5:**
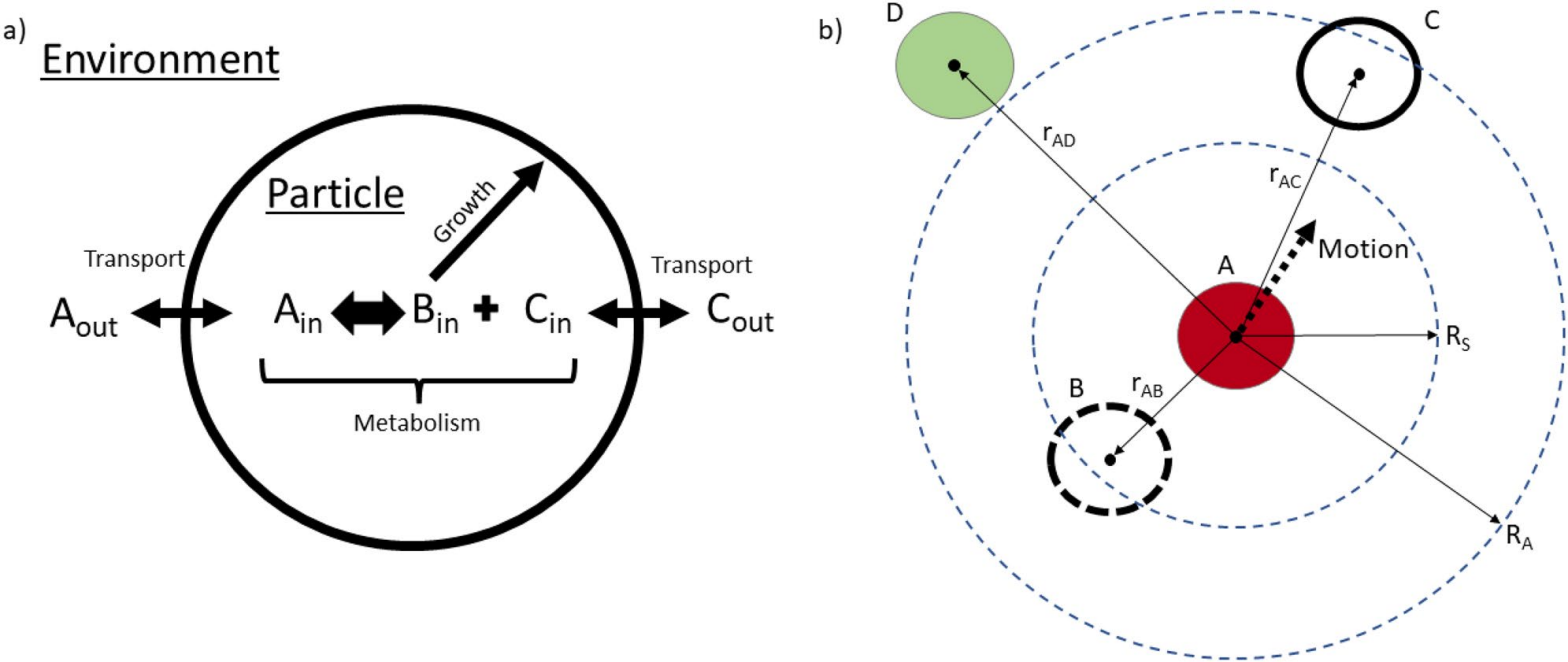
Cartoon showing the particle model and particle-particle interactions. (**a**) The particle contains an internal chemical system. Chemicals *A* and *C* are found in the environment and may be exchanged with the particle along the boundary surface. A simple metabolic model converts between *A* and coupled *B* and *C* while *B* is used to control the growth of the particle as described by Eq. 2. (**b**) Particles interact through soft force laws depending on the inter-particle distance. Particle A interact with both particle B and C while particle D is outside of the interaction distance limit, $r_{AD} > R_A$. For an example system where the particles have the same radii $R_S$ is the same. Pairing B and A experience a repulsive force as $r_{AB}<R_S$ and pairing C and A experience an attractive force as $R_S<r_{AC}<R_A$. The resulting force that motivates the motion of particle is in a direction towards C.

Particles in BMX are described by a set of physical properties and a cell metabolic model. Particles are considered as chemically active systems, separate from the domain, with a finite volume ${\mathscr {V}}_{particle}$ enclosed by a spherical boundary with surface area ${\mathscr {A}}_{particle}$. The chemical reactions within the particle are described by the cell metabolic model and chemical concentrations are exchanged between the particle system and the environment across the boundary, as shown in Figure 5. The metabolic model affects the volume and surface area of the particle which in turn affects the flux and exchange through the surface.

The cell metabolism model is provided by the user as a set of chemical reaction ODEs describing the changes in concentration of cell bound chemical species. In this article we considered a simple set of equations we refer to as the “ABC” metabolism within which three chemical constituents are considered; *A*, *B* and *C*. *A* and *C* exist both inside and outside the cell (in the agar medium) with *A* being a nutrient source and *C* a waste metabolic byproduct. The concentrations outside the particle are denoted with the subscript *out* and those inside the particle are labeled with the subscript *in*. The compound *B* is only located inside the particle and is used to control changes in particle size. These species are related to each other via the following reactions:

$$\begin{aligned}{} & {} \hbox {A}_{\textrm{out}} \mathop {\rightleftharpoons }\limits ^{k_1}_{k_{-1}} \quad \hbox {A}_{\textrm{in}} \\{} & {} \hbox {A}_{\textrm{in}} \mathop {\rightleftharpoons }\limits ^{k_2}_{k_{-2}} \quad \hbox {B}_{\textrm{in}} + \hbox {C}_{\textrm{in}} \\{} & {} \hbox {C}_{\textrm{in}} \mathop {\rightleftharpoons }\limits ^{k_3}_{k_{-3}} \quad \hbox {C}_{\textrm{out}} \end{aligned}$$

The rate constants for transport into and out of the particles are discussed in Supplementary Information S.3.

To distinguish between the concentration of a species and the absolute amount of a species in a grid cell or particle, the following notational convention is used. If the species is enclosed in square brackets, e.g. $[B_{in}]$, then it represents a concentration, if it is without brackets, $B_{in}$, then it refers to the absolute amount of the species.

An additional to the reactions listed above, a reaction is needed to couple the chemical reactions to cell growth. In the “ABC” model, the component *B* is used to build cell material so the rate of change of volume is proportional to the existing size of the cell, ${\mathscr {V}}_{particle}$, and the rate of change of *B*. This leads to the growth equation

2 $$\begin{aligned} \frac{d{\mathscr {V}}_{particle}}{dt} = k_g\frac{d[B_{in}]}{dt}{\mathscr {V}}_{particle} \end{aligned}$$

where $k_g$ is the proportionality constant. To capture the phenomena of bacteria cell division the particles divide into two particles, parent and offspring, when a threshold condition is set. Currently, a critical particle volume threshold ${\mathscr {V}}_{crit}$ is implemented such that when the particle volume is greater than the threshold, ${\mathscr {V}}_{particle}>{\mathscr {V}}_{crit}$, a new particle is introduced in a random orientation about the parent particle and the volume and chemical contents of the parent particle are shared between the parent and offspring. That is,

$$\begin{aligned} ([A]_{in}^{parent},[B]_{in}^{parent},[C]_{in}^{parent},{\mathscr {V}}^{parent}_{particle})&= ([A]_{in}/2,[B]_{in}/2,[C]_{in}/2,{\mathscr {V}}_{particle}/2) \\ ([A]_{in}^{offspring},[B]_{in}^{offspring},[C]_{in}^{offspring},{\mathscr {V}}_{particle}^{offspring})&= ([A]_{in}/2,[B]_{in}/2,[C]_{in}/2,{\mathscr {V}}_{particle}/2) \end{aligned}$$

with a re-calculation for the particles’ surface areas.

Particles are expected to move due to forces exerted on them. In BMX, particle interaction dynamics are produced by considering the exclusion volume between the particles. The force between two particles is calculated using a cubic interaction as described in Mathias *et al*^38^. The interactions are pairwise additive, with the force between two particles *i* and *j* described by a function of the form

$$\begin{aligned} {\overline{F}}_{ij} = F_{ij}(r_{ij})\frac{{\overline{r}}_{ij}}{r_{ij}} \end{aligned}$$

where $F_{ij}(r_{ij})$ is a spherically symmetric function of the separation distance between the centers of the two particles. For this model, the function *F*(*r*) has the generic form

$$\begin{aligned} F(r)= & {} -\mu (r-R_A)^2(r-R_S)\;\;\;\text{ if }\;\;\;r<R_A \\= & {} 0\;\;\; \text{ otherwise } \end{aligned}$$

The parameter $\mu$ is the stiffness and controls the magnitude of the repulsive and attractive interactions. $R_S$ is the equilibrium separation value at which the net force on the particles is zero and $R_A$ is the distance at which all pair interactions vanish. For $r<R_S$, the particles repel each other while for $R_S<r<R_A$, the particles are attracted to each other. For this model, the values of $R_S$ and $R_A$ are functions of the individual particle radii. If particle *i* has radius ${\mathscr {R}}_i$ and particle *j* has radius ${\mathscr {R}}_j$ then the parameter $R_S$ is chosen as

$$\begin{aligned} R_S = {\mathscr {R}}_i+{\mathscr {R}}_j \end{aligned}$$

The width of the attractive region, $R_A-R_S$, is chosen to have a fixed value $W_A$ for all particle interactions. The value of $R_A$ is then

$$\begin{aligned} R_A = {\mathscr {R}}_i+{\mathscr {R}}_j +W_A \end{aligned}$$

Note that the particle radii can increase over time, so even if two particles are in mechanical equilibrium at some time *t*, they may start pushing against each other as they grow.

In addition to the particle-particle interactions, particles also interact with the surface of the growth medium at the air-agar interface using a model similar to the particle-particle interaction. For this interaction, the contribution to the force is only in the *z*-direction and is a function *H*(*z*) of the height *z* above the growth medium surface. The force has the form

$$\begin{aligned} H(z)= & {} -\xi (z-Z_A)^2(z-Z_S)\;\;\;\text{ if }\;\;\;0<z<Z_A \\= &\, \xi Z_A^2Z_S\;\;\;\text{ if }\;\;\;z<0 \\= &\, 0\;\;\;\text{ otherwise } \end{aligned}$$

where the parameter $\xi$ is the stiffness of the interaction, $Z_S$ is the equilibrium distance above the surface and $Z_A$ is the point at which all interaction with the surface ends. Between $Z_S<z<Z_A$, particles are attracted to the surface, while below $Z_S$ particles are repelled from the surface. Below the surface, particles experience a constant upward force. This force is designed primarily to prevent particles from accidentally getting pushed down into the growth medium. Similar to the pair interaction, for particle *i*, the parameter $Z_S$ is given by

$$\begin{aligned} Z_S = {\mathscr {R}}_i \end{aligned}$$

and the parameter $Z_A$ is given by

$$\begin{aligned} Z_A = {\mathscr {R}}_i + W_Z \end{aligned}$$

where $W_Z$ is the width of the attractive region and is assumed to be independent of the particle’s size.

## Supplementary Information

Supplementary Information.

## Data Availability

The datasets generated and/or analysed during the current study are available in the zenodo repository, *10.5281/zenodo.8084270*.
