# Supplementary material for: BMX: Biological modelling and interface exchange
Source: Sci Rep. 2023 Jul 28;13:12235. doi: 10.1038/s41598-023-39150-1 (PMC10382537; doi:10.1038/s41598-023-39150-1)
Supplement: Supplementary file 1 — Supplementary Information. [file 41598_2023_39150_MOESM1_ESM.pdf]

Supplementary information for: BMX: Biological modelling and interface exchange

Bruce J. Palmer[1], Ann S. Almgren[2], Connah G. M. Johnson[1,*], Andrew T. Myers[2], and William R. Cannon[1]

[1]Pacific Northwest National Laboratory, Washington, USA
[2]Lawrence Berkeley National Laboratory, Berkeley, California, USA
[*]Corresponding author connah.johnson@pnnl.gov

S.1 High performance scaling

The focus of the main article was to develop an efficient computational framework for simulating the growth of large numbers of cells coupled to an environment in which the transport of nutrients is realistically modeled. The calculations presented here are relatively small compared to those performed using the AMReX framework in other application areas[1,2], but they still benefit from the use of multiple processors and GPUs. Some short timing runs of 1000 steps were done using the final configuration from the run shown in Figure 1 in the main article as the initial condition. This configuration was chosen as the starting point since it represents a mix of both calculation components, the transport calculations on the grid and the particle calculations. Timings for the original initial condition with only one particle would be more heavily biased towards the grid. Speedup on p processes is defined as $S_p = \tau_1/\tau_p$, where τ_p is the amount of time required for the code to run using p processes. The speedup on one process is by definition equal to 1. Perfect scaling is defined as $S_p = p$, implying that a calculation run on p processes requires $1/p$ the amount of time as a calculation run on 1 process.

The partitioning of work between the grid and particle parts of the calculation shows more variation. The amount of time spent updating mesh values (dominated by chemical species diffusion and chemical reactions) and computing the particle forces and position updates is shown in Figure S.1-a for the regular cluster. The amount of time spent evaluating particle updates is initially quite small, reflecting the small number of particles, but grows larger as the number of particles increases and surpasses the amount of time spent spent updating the grid at around 70000 seconds. The discontinuity at 200000 seconds in Figure S.1-a occurs at a point where the calculation was restarted (at a later date) from a previous calculation and probably represents some changes in computer system load and possibly system configuration. The corresponding curves for the irregular cluster are relatively flat, reflecting the smaller number of particles generated by this calculation and the fact that the grid calculation consistently requires more time. As high performance is a key aim for BMX the speed up scaling with the number of processors was considered. Figure S.1-b shows a strong scaling study of the system in Figure 1, based on runs using from 1 to 64 processors using only CPUs. Almost ideal scaling is shown from 1 to 8 cores; slightly less than ideal scaling occurs from 8 to 64 cores but this is not surprising given the relative smallness of the system.

BMX is capable of supporting simulations with large number of bacteria cell particles as shown in Figure S.3. Here, the simple "ABC" metabolic model was run on slightly thinner system by with much larger extent in the x and y directions. The dimensions are $512 \times 512 \times 16$ μm with the agar surface at 8 μm. This configuration was simulated for a longer time span showing a linear colony growth over time. The agar support appears to be deep enough to ensure that the bacteria cells are not starved of nutrients and produce the smooth boundary similar to that seen in Figure 1-a. The total number of particles at the end of the simulation is 37598.

BMX can utilize GPUs, when available. Figure S.2 shows two comparisons between a calculation run using CPUs and GPUs. As in Figure S.1-b, the simulations were performed on the last configuration of the simulation shown in Figure 1. The CPU calculation shows good scaling over the limited number of processors shown in the plot, but it does not outperform the GPUs which are considerably faster, even using just using 1 GPU. For this sized calculation, the GPU performance appears to have reached a maximum at 1 GPU and no further gains are achieved by going to larger numbers of GPUs. Other calculations have shown some speedup in utilizing multiple GPUs. The comparison between GPU and CPU performance can be more complicated than shown here in that typically resources are allocated on parallel computers one node at a time. Since a node usually contains many more CPUs than GPUs, it is possible that using all the CPUs on a node may outperform the more limited number of GPUs. However, on these calculations it was found that using the optimum number of CPUs on a node still lead to

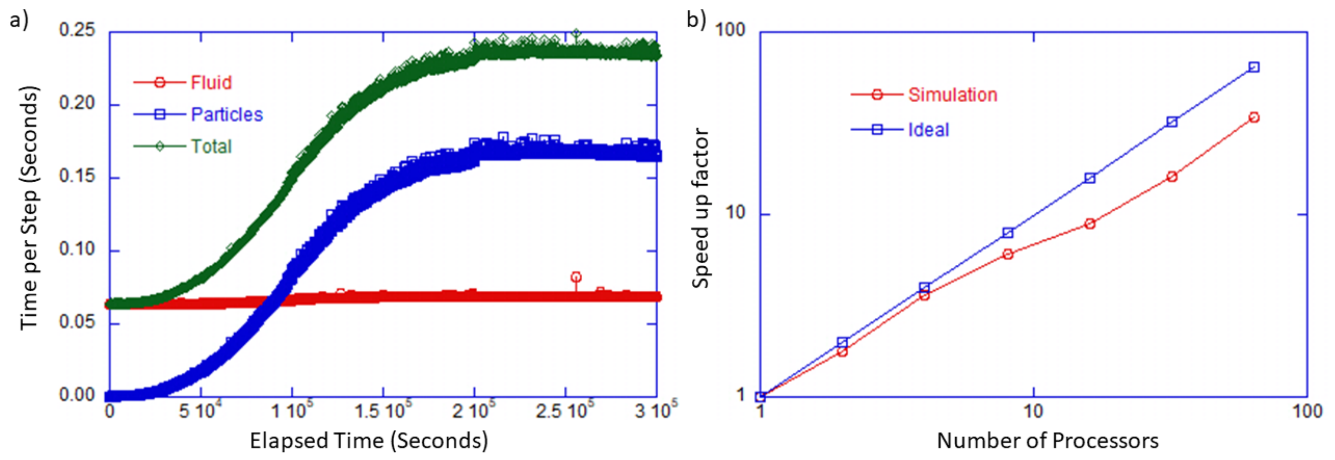

Figure S.1. The simulation time scales with the number of particles in the simulation and can be sped up by using more processors. a) Amount of time spent in updating grid (fluid) and particle per time step over the course of the regular cluster calculation showed in Figure 1. A slight discontinuity may be seen at 200000 seconds due to a checkpoint restart. b) Log-log plot of speedup curve for system shown in Figure 1. The curve for ideal speedup is included for reference.

lower performance than using the optimum number of GPUs.

It is also instructive to compare the performance of the two main components of the simulation, the computation on the grid and the computation over particles for GPUs and CPUs. Figure S.2-b shows the amount of time per time step spent in the grid and particle updates. For calculations done on the CPU, the two components are roughly comparable, the particle updates taking less than twice the time spent in the grid updates. On GPUs, however, the particle updates are nearly an order of magnitude faster than the grid updates. Although the chemistry model in the particles is extremely simple, these results suggest that the BMX framework will be able to support much more complicated chemistry inside the particles, provided it can be kept on the GPUs.

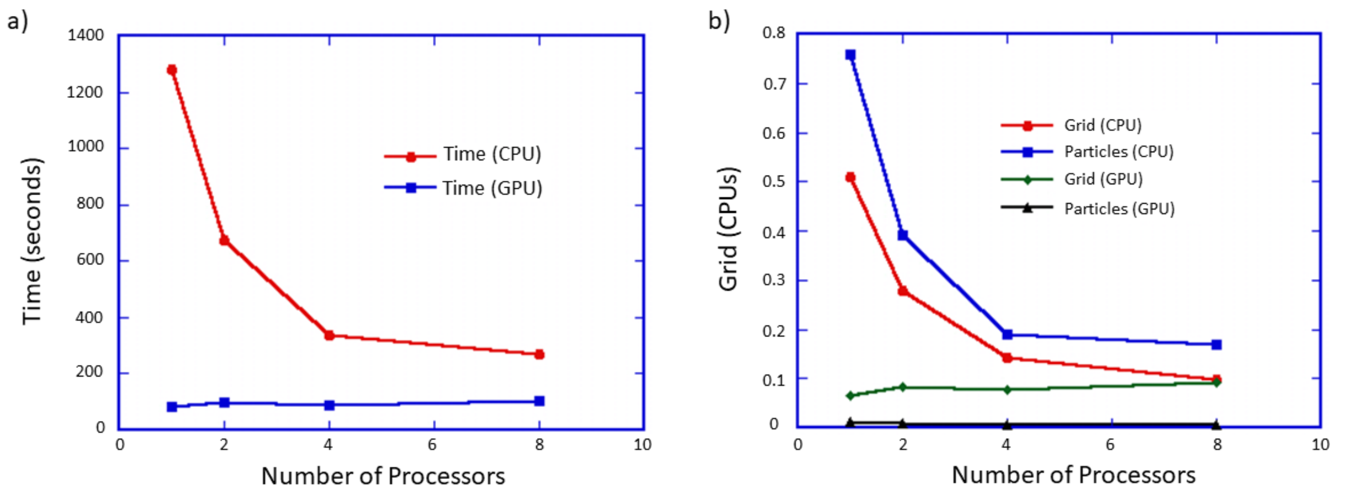

Figure S.2. Simulations performed on GPU processors outperform simulations performed on CPU processors. a) Comparison of speedup curves for the system shown in Figure 1 when running on CPUs and GPUs. b) Comparison of time per step for calculating the grid and particle components of the system for the simulation shown in Figure 1 when running on CPUs and GPUs.

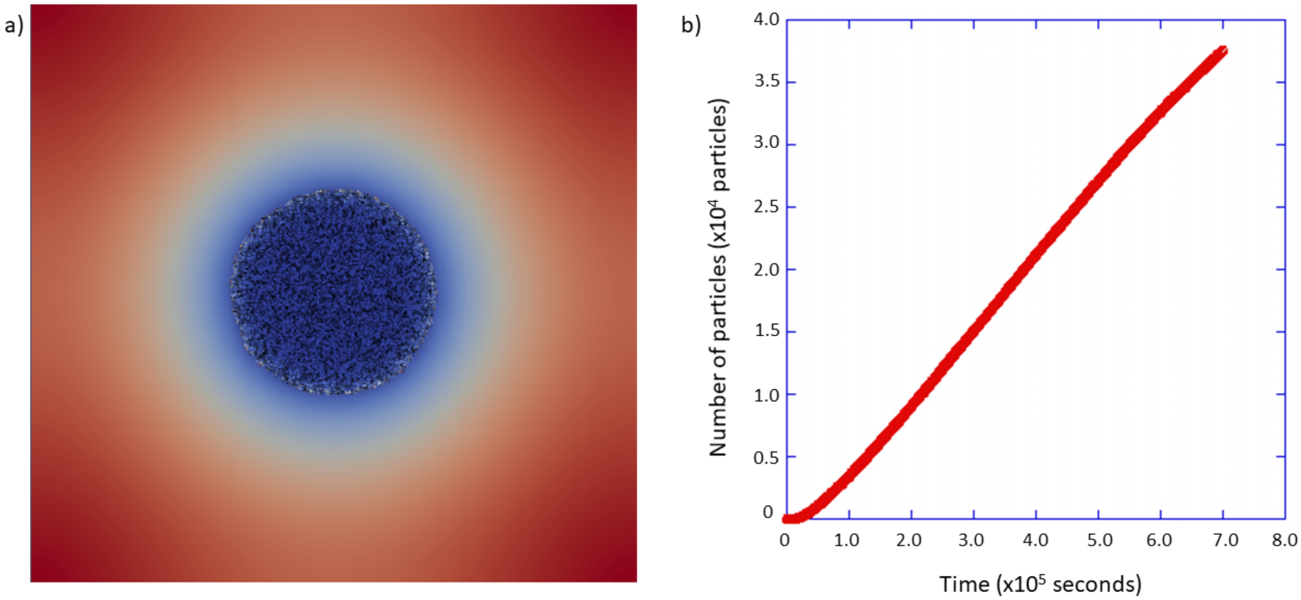

Figure S.3. Long time simulation of a growing cell colony. a) A top-down view of the simulation domain showing the growing colony. b) The number of bacteria particles in the colony over time.

S.2 Transport Model

As discussed in the main article, the transport of nutrients through the nutrient medium is governed by the diffusion equation

$$\frac{dc_i}{dt} = -D\nabla c_i + S_i(\mathbf{C}) \tag{1}$$

where the symbols were defined previously in the main article. Based on earlier work by Weissberg[3], Khirevich *et al.* tried mapping their results for diffusion in packed beds using the following form for the tortuosity

$$\tau = 1 - p\ln\phi$$

where p is an adjustable parameter. Weissberg originally proposed a value of 0.5 for p. Depending on the details of the packing algorithm used to create the packed bed, Khirevich *et al.* found values of p that varied from 0.43 to 0.5. They also found fairly high values of R^2 for their fits. From this they concluded that this form of the tortuosity was insufficient for their purposes (modeling chromatography columns). However, given the uncertainties in modeling biological systems, we feel that this form is accurate enough to model extracellular diffusion. We used a value of 0.45 for p.

A problem with this formula is that it is generally possible for the volume fraction of fluid in the grid cell to go to zero, resulting in negative or extremely small diffusion coefficients. This is undesirable, since it completely shuts off transport of nutrients into and out of the particles in this grid cell from the surrounding fluid. The particle stops growing, even under circumstances where we would expect that nutrients could still get to the particle through mechanisms such as diffusion through a thin fluid film on the cell surface or diffusion through other cells. To prevent unphysical low diffusion, $D_{eff,i}$ is not allowed to drop below $0.1D_{0_i}$.

S.3 Particle Model

The particles in the simulations described here each possess a simple "ABC" metabolic model. The chemical species in the metabolism are related to each other via the following reactions:

$$\mathrm{A_{out}} \underset{k_{-1}}{\overset{k_1}{\rightleftharpoons}} \mathrm{A_{in}}$$

$$\mathrm{A_{in}} \underset{k_{-2}}{\overset{k_2}{\rightleftharpoons}} \mathrm{B_{in}} + \mathrm{C_{in}}$$

$$\mathrm{C_{in}} \underset{k_{-3}}{\overset{k_3}{\rightleftharpoons}} \mathrm{C_{out}}$$

As described previously, to distinguish between concentrations of these components and absolute amounts of the component, we use brackets [ ]. In this notation, $[A_{out}]$ is the concentration of A in a grid cell and A_{out} is the total amount (mass) of A in a grid cell. The concentrations of these species are governed by the set of equations

$$\begin{aligned}
\frac{d[A_{out}]}{dt} &= -k_1[A_{out}] + k_{-1}[A_{in}] \\
\frac{d[A_{in}]}{dt} &= k_1[A_{out}] - k_{-1}[A_{in}] - k_2[A_{in}] + k_{-2}[B_{in}][C_{in}] \\
\frac{d[B_{in}]}{dt} &= k_2[A_{in}] - k_{-2}[B_{in}][C_{in}] \\
\frac{d[C_{in}]}{dt} &= k_2[A_{in}] - k_{-2}[B_{in}][C_{in}] - k_{-3}[C_{in}] + k_3[C_{out}] \\
\frac{d[C_{out}]}{dt} &= k_{-3}[C_{in}] - k_3[C_{out}]
\end{aligned}$$

These reactions also act as sources and sinks for the diffusion equations governing transport of A_{out} and C_{out} in the agar growth medium.

These coupled rate equations could be handled by a time integration package but we chose to implement a simple integration scheme for two reasons. The first is that given the data layout in AMReX and their model for interacting to GPUs it would be difficult or impossible to incorporate a library-base time integration package into the code. The second reason is that it is not clear that the concentration positivity constraints, discussed in the next section, for concentrations both in the cell and in the fluid could be easily enforced using a standard integrator.

The mass flux equations into and out of the particle can be examined in more detail. The particle membrane is permeable only to the chemicals A and C and we define coupled coefficients (k'_1, k'_{-1}) and (k'_3, k'_{-3}) as mass-transfer coefficients for transport of A and B, respectively. The fluxes of material into the cell, f_{in}, and out of the cell, f_{out}, can be written as

$$\begin{aligned}
f^A_{in} &= [A_{out}]\mathscr{A}_{particle}k'_1 \\
f^A_{out} &= [A_{in}]\mathscr{A}_{particle}k'_{-1} \\
f^C_{in} &= [C_{out}]\mathscr{A}_{particle}k'_3 \\
f^C_{out} &= [C_{in}]\mathscr{A}_{particle}k'_{-3}
\end{aligned}$$

In a time increment Δt, the chemical mass increments ΔA_{in} and ΔC_{in} going into the particle and ΔA_{out} and ΔC_{out} going out of the particle are

$$\begin{aligned}
\Delta A_{in} &= \Delta t \frac{A_{out}}{\mathscr{V}_{grid}}\mathscr{A}_{particle}k'_1 \\
\Delta A_{out} &= \Delta t \frac{A_{in}}{\mathscr{V}_{particle}}\mathscr{A}_{particle}k'_{-1} \\
\Delta C_{in} &= \Delta t \frac{C_{out}}{\mathscr{V}_{grid}}\mathscr{A}_{particle}k'_3 \\
\Delta C_{out} &= \Delta t \frac{C_{in}}{\mathscr{V}_{particle}}\mathscr{A}_{particle}k'_{-3}
\end{aligned}$$

Note that $(A_{in}, A_{out}, C_{in}, C_{out})$ are the absolute amounts of the chemicals occupying a particle volume $\mathscr{V}_{particle}$ and fluid volume in a grid cell $\mathscr{V}_{grid}$, respectively. We are ignoring possible changes in the particle and grid cell volumes during this time interval. The incremental changes in the total amount of material in the particle and the grid cells can be written as

$$\begin{aligned}
A_{in}(t+\Delta t) - A_{in}(t) &= \Delta t \frac{A_{out}}{\mathscr{V}_{grid}}\mathscr{A}_{particle}k'_1 - \Delta t \frac{A_{in}}{\mathscr{V}_{particle}}\mathscr{A}_{cell}k'_{-1} \\
A_{out}(t+\Delta t) - A_{out}(t) &= \Delta t \frac{A_{in}}{\mathscr{V}_{particle}}\mathscr{A}_{particle}k'_{-1} - \Delta t \frac{A_{out}}{\mathscr{V}_{grid}}\mathscr{A}_{particle}k'_1 \\
C_{in}(t+\Delta t) - C_{in}(t) &= \Delta t \frac{C_{out}}{\mathscr{V}_{grid}}\mathscr{A}_{cell}k'_3 - \Delta t \frac{C_{in}}{\mathscr{V}_{particle}}\mathscr{A}_{particle}k'_{-3} \\
C_{out}(t+\Delta t) - C_{out}(t) &= \Delta t \frac{C_{in}}{\mathscr{V}_{particle}}\mathscr{A}_{particle}k'_{-3} - \Delta t \frac{C_{out}}{\mathscr{V}_{grid}}\mathscr{A}_{particle}k'_3
\end{aligned}$$

Dividing the first equation by $\Delta t \mathscr{V}_{cell}$ and the second by $\Delta t \mathscr{V}_{grid}$ and taking the limit $\Delta t \to 0$ gives

$$
\begin{aligned}
\frac{d[A_{in}]}{dt} &= [A_{out}]\frac{\mathscr{A}_{particle}}{\mathscr{V}_{particle}}k_1' - [A_{in}]\frac{\mathscr{A}_{particle}}{\mathscr{V}_{particle}}k_1' \\
\frac{d[A_{out}]}{dt} &= [A_{in}]\frac{\mathscr{A}_{particle}}{\mathscr{V}_{grid}}k_{-1}' - [A_{out}]\frac{\mathscr{A}_{particle}}{\mathscr{V}_{grid}}k_1' \\
\frac{d[C_{in}]}{dt} &= [C_{out}]\frac{\mathscr{A}_{particle}}{\mathscr{V}_{particle}}k_3' - [C_{in}]\frac{\mathscr{A}_{particle}}{\mathscr{V}_{particle}}k_3' \\
\frac{d[C_{out}]}{dt} &= [C_{in}]\frac{\mathscr{A}_{particle}}{\mathscr{V}_{grid}}k_{-3}' - [C_{out}]\frac{\mathscr{A}_{particle}}{\mathscr{V}_{grid}}k_3'
\end{aligned}
$$

Using these expressions for the change in concentration due to transport across the cell membrane, the rate equations governing this system become

$$
\begin{aligned}
\frac{d[A_{out}]}{dt} &= -k_1'\frac{\mathscr{A}_{particle}}{\mathscr{V}_{grid}}[A_{out}] + k_{-1}'\frac{\mathscr{A}_{particle}}{\mathscr{V}_{grid}}[A_{in}] \\
\frac{d[A_{in}]}{dt} &= k_1'\frac{\mathscr{A}_{particle}}{\mathscr{V}_{particle}}[A_{out}] - k_{-1}'\frac{\mathscr{A}_{particle}}{\mathscr{V}_{particle}}[A_{in}] - k_2[A_{in}] + k_{-2}[B_{in}][C_{in}] \\
\frac{d[B_{in}]}{dt} &= k_2[A_{in}] - k_{-2}[B_{in}][C_{in}] \\
\frac{d[C_{in}]}{dt} &= k_2[A_{in}] - k_{-2}[B_{in}][C_{in}] - k_{-3}'\frac{\mathscr{A}_{particle}}{\mathscr{V}_{particle}}[C_{in}] + k_3'\frac{\mathscr{A}_{particle}}{\mathscr{V}_{particle}}[C_{out}] \\
\frac{d[C_{out}]}{dt} &= k_{-3}'\frac{\mathscr{A}_{particle}}{\mathscr{V}_{grid}}[C_{in}] - k_3'\frac{\mathscr{A}_{particle}}{\mathscr{V}_{grid}}[C_{out}]
\end{aligned}
$$

From here on in, we drop the primes on k_1', k_{-1}', k_3' and k_{-3}'.

Numerically, the rate equations are currently being handled using the following scheme:

- There is an equilibrium between the concentration of species in the fluid and the particle over a time increment $\Delta t/2$
- The internal particle reactions take place over a time increment Δt using the updated internal concentrations from the previous step. The cell volume is also updated during this step
- Using the updated internal concentrations from the reaction step, the concentrations between the inside and outside fluids are re-equilibrated over a time interval $\Delta t/2$

The first step is implemented by calculating the amount of material that flows between the particle and the grid cell in which it is located. The total amount of material that flows into the cell in the interval Δt is

$$
\begin{aligned}
\Delta A &= \frac{\Delta t}{2}\mathscr{A}_{particle}(k_1[A_{out}] - k_{-1}[A_{in}]) \\
\Delta B &= 0 \\
\Delta C &= \frac{\Delta t}{2}\mathscr{A}_{particle}(k_3[C_{out}] - k_{-3}[C_{in}])
\end{aligned}
$$

Based on this increment, the concentrations inside and outside the particle are adjusted using the equations

$$
\begin{aligned}
[A_{in}'] &= [A_{in}] + \Delta A/\mathscr{V}_{particle} \\
[B_{in}'] &= [B_{in}] \\
[C_{in}'] &= [C_{in}] + \Delta C/\mathscr{V}_{particle}
\end{aligned}
$$

$$
\begin{aligned}
[A_{out}'] &= [A_{out}] - \Delta A/\mathscr{V}_{grid} \\
[B_{out}'] &= [B_{out}] \\
[C_{out}'] &= [C_{out}] - \Delta C/\mathscr{V}_{grid}
\end{aligned}
$$

The second step adjusts the concentration of reactants inside the particle based on internal chemical reactions. Using a simple Euler scheme, the adjusted values of the concentrations and the particle volume after the time increment Δt are

$$\begin{aligned}
[A'_{in}] &= [A_{in}] + \Delta t(-k_2[A_{in}] + k_{-2}[B_{in}][C_{in}]) \\
[B'_{in}] &= [B_{in}] + \Delta t(k_2[A_{in}] - k_{-2}[B_{in}][C_{in}]) \\
[C'_{in}] &= [C_{in}] + \Delta t(k_2[A_{in}] - k_{-2}[B_{in}][C_{in}]) \\
\mathscr{V}'_{particle} &= \mathscr{V}_{particle} + dt(k_g([A_{in}] - k_{-2}[B_{in}][C_{in}])\mathscr{V}_{particle}
\end{aligned}$$

After adjusting the volumes, the concentrations need to be modified slightly by multiplying by the ratio of $\mathscr{V}_{particle}$ and $\mathscr{V}'_{particle}$

$$\begin{aligned}
[A''_{in}] &= [A'_{in}]\frac{\mathscr{V}'_{particle}}{\mathscr{V}_{particle}} \\
[B''_{in}] &= [B'_{in}]\frac{\mathscr{V}'_{particle}}{\mathscr{V}_{particle}} \\
[C''_{in}] &= [C'_{in}]\frac{\mathscr{V}'_{particle}}{\mathscr{V}_{particle}}
\end{aligned}$$

The concentrations inside and outside the particle are then adjusted one more time using the equations in the first step.

The increments in A and C between the exterior fluid and the particle interior from the first and last step are added together to give a total increment between the fluid and the particle interior. At the end of the step, the increment is divided by the fluid volume, $\mathscr{V}_{grid}$, in the grid cell to get the change in concentration in the grid cell hosting the particle.

S.4 Maintaining Non-negative Concentrations

Chemical concentrations are positive quantities. Therefore an important part of successfully running a simulation is to guarantee that at no time do the concentrations of any chemical species fall below zero, in either the particle or in the grid cell. This can occur because the finite value of the time increment can cause values to overshoot their true values by a small amount, resulting in negative concentrations after exchanges. The potential for error is amplified when considering grid volume that contain multiple particles, each simultaneously adding or subtracting from the grid concentrations. This can be rectified by using a smaller time step, but this is also undesirable since it results in longer simulation times with relatively little change in physical behavior. A better approach is to modify the algorithm to correct for negative concentrations when they occur.

The BMX strategy for maintaining positive concentrations consists of two parts. The first is to divide the available fluid in each grid cell evenly between each of the particles located in the grid cell and the second is to check if any of the components in the reaction schemes inside the particles goes negative. Apportioning the fluid volume evenly between the particles is designed to handle the issue that the chemistry of multiple particles can potentially interact by exchanging material through the fluid that they all share in common. However, a chemical integration scheme that updates particles individually will not account for this and while each individual particle may be able to maintain non-negative concentrations, multiple particles acting independently of each other in a single grid cell could result in a negative fluid concentration. If the fluid is divided up between the each particle in the grid cell, then this cannot happen if the chemistry of the individual particles has been implemented to guarantee that the internal concentration and fluid concentrations all remain positive.

Concentrations inside the particle or in the fluid can go negative because a finite time step causes the system to overshoot a limit on the available reactant. If this occurs, then the change in that component is modified so that the component only goes to zero. This means that the change in any component that is coupled to the changed component must also be adjusted. For example, for the reaction

$$\mathrm{A} \longrightarrow \mathrm{B} + \mathrm{C}$$

we might calculate a change in A over one time step, ΔA, such that A goes negative. This can be corrected by decreasing the change in A to a value $\Delta A'$ so that A goes to zero instead. However, this means that the changes in B and C must also be modified to reflect the smaller change in A.

S.4.1 Parameters

In this article we have viewed the particles as representing bacteria cells utilising a simple "ABC" metabolic model. Even with the simple "ABC" model presented here, this model has a substantial number of parameters that need to be set in order for the simulation to produce meaningful results. For the purposes of this study, the parameters were chosen to give results comparable to the behavior of a typical bacterial colony with the qualitative results being compared to literature and

experimental observations. The parameters used were sufficient to demonstrate that the growth model can be handled by the existing numerical framework and integration schemes introduced within BMX. We expect that a more realistic metabolism model would be characterized by many more reactions and parameters with increased simulation complexity, but from a numerical point of view would not be qualitatively different from the model presented here.

The volume of a bacteria cell that is 1 μm in diameter is 5.3×10^{-13} cm^3 so the volume threshold for splitting into 2 cells, $\mathscr{V}_{crit}$, was set at approximated twice that value, 1×10^{-12} cm^3 (the radius at splitting is about 6.2 μm). Given that the dimension of individual cells are in the range of 1 μm in diameter, a minimum grid cell size was also set to 1 μm. The remaining parameters were chosen so that particle division occurred on a time scale of 30 minutes to an hour. The diffusion coefficient for a nutrient molecule such as glucose is approximately 600 cm^2/s and this was used as the diffusion coefficient for both D_A and D_C in the extracellular medium. The initial concentration of A was chosen to be 20 mMolar. The concentration of C was chosen to have a non-zero value at the beginning of the simulation and was arbitrarily set to 2 mMolar. The component B is only found inside the bacteria cell particles and its concentration in the extracellular medium is zero.

The rate constants were chosen through a combination of analysis and empirical adjustment to give the desired behavior. Values were adjusted to give a timescale of approximately 30 minutes to an hour for cell division. For a realistic model, these parameters would be known, or mostly known, *a priori* and would not be subjected to additional adjustment. The complete set of parameters is summarized in Table S.1.

Variable	Value	Description
Δt	1 sec	Time increment
Δx	1×10^{-4} cm	Minimum grid cell dimension
$\mathscr{V}_{crit}$	1.0×10^{-12} cm^3	Volume at which cell divides
$[A_{out}]_0$	2.0×10^{-5} M/cm^3	Initial external concentration of A
$[C_{out}]_0$	2.0×10^{-6} M/cm^3	Initial external concentration of C
D_A	6×10^{-10} cm^2/sec	Diffusion coefficient of A
D_C	6×10^{-10} cm^2/sec	Diffusion coefficient of C
k_1	2.0×10^{-5} cm/sec	Reaction coefficient
k_{-1}	2.0×10^{-5} cm/sec	Reaction coefficient
k_2	0.4 sec^{-1}	Reaction coefficient
k_{-2}	0.06moles/(cm^3 sec)	Reaction coefficient
k_3	2.0×10^{-5} cm/sec	Reaction coefficient
k_{-3}	2.0×10^{-5} cm/sec	Reaction coefficient
k_g	1000 cm^3/moles	Growth rate coefficient

Table S.1. Summary of parameters used in bacteria cell growth and metabolism model

S.5 Installation and Usage Instructions

BMX has been developed to provide a portable high-performance agent-based simulation suite for large scale cell community modelling. The portable nature of BMX means that the software can be installed and run on a range of different hardware architectures with minimal user intervention. One of the advancing capabilities of BMX is the GPU acceleration which is shown in Section 1 to greatly improve the computation speed for large cell community simulations. A GPU is not however a requirement to run BMX and good simulation speeds can be achieved through CPU computation alone. Therefore, BMX can be readily used across a range of computing systems and as such provides an accessible suite for large scale simulations.

Adaptability was a design aim for BMX allowing a range of biological problems to be considered. A user's simulation may be implemented by modifying the input files and by editing the chemical evolution routines of the c++ source code. The input files are used to determine both the solver/simulation settings, to provide the rate parameters for chemical reactions, and input the simulation initial conditions. The simulation outputs are provided as standardized *.vtk* files for rapid post-processing by a user's preferred visualization software.

S.5.1 Installation

The source code for BMX is available from the *DOI:10.5281/zenodo.8084270* zenodo repository (under the BSD-2-Clause licence). After downloading and extracting the source code the suite can be setup through navigating to the top directory and installing the AMReX submodule:

1. Remove the existing AMRex directory: *rm -rf subprojects/amrex*

2. Initializing the submodule: *git submodule init*
3. Updating the AMReX submodule: *git submodule update*

After the AMReX submodule setup BMX can be configured and run. To configure BMX for a first time installation, from the top directory:

1. Make a separate build directory: *mkdir build*
2. Navigate to the build directory: *cd build*
3. cmake the software code: *cmake ..*
4. Compile the executable: *make*
5. Navigate to the desired simulation directory (e.g the *nlev_large_test* simulation): *cd ../exec/nlev_large_test/*
6. From the simulation directory run the built executable with the simulation file as input: *../../build/bmx input_real_nlev*

For subsequent simulation runs the procedure varies depending on whether the c++ source code is modified. If the code has been modified (e.g for implementing different chemical systems), from the top directory, step 1 can be omitted but the cmake command and beyond are still required. If only the simulation input files are modified then steps 1 to 4 can be omitted.

S.5.2 Implementing a User Simulation

To run a BMX, a simulation input file is required. A typical input file describes the solver settings, Input-Output and check-pointing settings, Fluid and chemical model parameters, cell-cell interaction force settings, and provides a file describing the cell particle initial conditions. An exemplar input file can be found at */exec/nlev_dla_test/input_ml2* which implements the "ABC" diffusion limited aggregation simulation whose parameters are described in Table S.1. This simulation input refers to a particle data file "abc_real_input2.dat" which describes the initial location of a single particle as the initial condition used to produce the figures in the manuscript. Should a greater number of initial particle be required then new lines can be added to the particle data file for each new particle and the first line value updated to reflect the new initial cell number. The settings in the input and particle files can be freely changed by a user to generate new simulation output.

In addition to modifying the simulation inputs, a bespoke simulation can be produced by altering the software code directly. The files most conducive for an effective tailored simulation may be found in the */src/chemistry/* directory. In particular, the particle-particle and particle-surface interactions can be modified in */src/chemistry/bmx_cell_interaction_K.H* and chemical reactions and growth rate equations can be modified in */src/chemistry/bmx_chem_K.H*. Should new inputs be required for the modified functions then they can be read in by modifying the */src/chemistry/bmx_cell_interaction.cpp* and */src/chemistry/bmx_chem.cpp* files respectively, with the associated values written in and read from the simulation input file.

S.5.3 Processing Simulation Output

The output from a BMX simulation is place within the simulation directory from which the simulation was executed (e.g */exec/nlev_large_test/*). The frequency at which the simulation data is written to the output files is specified within the simulation input file and for each output time step a new *.vtk* file is created. These files contain the information for both the fluid domain concentration data and the cell particle data. While a user can utilize a preferred post-processing or visualization software ParaView[4] is recommended. In ParaView, the fluid concentration fields load the *plt* files through the provided "AMReX/BoxLib Grid Reader" reader and selecting the "X_I" cell array value for the concentration of chemical "I". To read in the particle data, use the provided "AMReX/BoxLib Particles Reader" reader. Different values can be input by selecting the "I" point array value for the internal particle concentration of chemical "I" alongside other desired values such as "cell_volume". Then the particles may be visualized by using the "Glyph" filter mode.

References

1. Kanagarajan, B., Quinlan, J. M. & Runnels, B. A diffuse interface method for solid-phase modeling of regression behavior in solid composite propellants. *Combust. Flame* **242** (2022).

2. Zeng, Y., Xuan, A., Blaschke, J. & Shen, L. A parallel cell-centered adaptive level set framework for efficient simulation of two-phase flows with subcycling and non-subcycling. *J. Comput. Phys.* **448** (2022).

3. Weissberg, H. L. Effective diffusion coefficient in porous media. *J. Appl. Phys.* **34**, 2636–2639 (1963). URL https://doi.org/10.1063/1.1729783. DOI 10.1063/1.1729783. https://doi.org/10.1063/1.1729783.

4. Ahrens, J., Geveci, B. & Law, C. ParaView: An end-user tool for large data visualization. *The visualization handbook* **717** (2005).
